# Unique evolution of foraminiferal calcification to survive global changes

**DOI:** 10.1126/sciadv.add3584

**Published:** 2023-06-21

**Authors:** Yurika Ujiié, Yoshiyuki Ishitani, Yukiko Nagai, Yoshihiro Takaki, Takashi Toyofuku, Shun’ichi Ishii

**Affiliations:** ^1^ Marine Core Research Institute, Kochi University, Kōchi, Japan.; ^2^ Institute for Extra-cutting-edge Science and Technology Avant-garde Research (X-star), Japan Agency for Marine-Earth Science and Technology (JAMSTEC), Yokosuka, Japan.; ^3^ National Museum of Nature and Science, Tokyo, Japan.; ^4^ Tokyo University of Marine Science and Technology (TUMSAT), Tokyo, Japan.

## Abstract

Foraminifera, the most ancient known calcium carbonate–producing eukaryotes, are crucial players in global biogeochemical cycles and well-used environmental indicators in biogeosciences. However, little is known about their calcification mechanisms. This impedes understanding the organismal responses to ocean acidification, which alters marine calcium carbonate production, potentially leading to biogeochemical cycle changes. We conducted comparative single-cell transcriptomics and fluorescent microscopy and identified calcium ion (Ca^2+^) transport/secretion genes and α-carbonic anhydrases that control calcification in a foraminifer. They actively take up Ca^2+^ to boost mitochondrial adenosine triphosphate synthesis during calcification but need to pump excess intracellular Ca^2+^ to the calcification site to prevent cell death. Unique α-carbonic anhydrase genes induce the generation of bicarbonate and proton from multiple CO_2_ sources. These control mechanisms have evolved independently since the Precambrian to enable the development of large cells and calcification despite decreasing Ca^2+^ concentrations and pH in seawater. The present findings provide previously unknown insights into the calcification mechanisms and their subsequent function in enduring ocean acidification.

## Teaser

Diversified Ca^2+^-related genes enable Rotaliida foraminifers to develop a unique strategy for surviving ocean acidification.

## INTRODUCTION

Calcifying marine organisms consume inorganic carbon dissolved in seawater, thereby contributing to the downward transport and long-term burial of carbon in the oceanic sinking of CO_2_. Climate change in the form of global warming and ocean acidification has severe consequences for biotic calcification due to lower seawater pH and carbonate saturation, leading to a possible collapse of marine ecosystems (*1*). To predict the impact of environmental change on biotic calcification, its mechanism and interaction with the physicochemical characteristics of seawater should be explored. Foraminifera account for ~25% of calcium carbonate (CaCO_3_) production in the present oceans because of their abundance in both seafloor and pelagic environments (*2*). Rotaliida, which includes benthic and all living planktonic species as the major foraminiferal order in the class Globothalamea, has been widely used in geological studies as a proxy for modern and paleo-environments, because the chemical and isotopic composition of its CaCO_3_ tests [e.g., magnesium/calcium (Mg/Ca) ratio as well as stable oxygen and carbon isotopes: δ^18^O and δ^13^C] is influenced by physicochemical conditions, such as the temperature and salinity of ambient seawater (*3*–*5*). Knowledge of major variations in climate through geological time derives predominantly from the analysis of foraminiferal tests. However, the nonequilibrium state of chemical compounds between foraminiferal tests and the external environment poses a problem for such measurements, because the uptake of Ca^2+^ and inorganic carbon is dictated by environmental conditions, as well as organisms, through the so-called “vital effect” (*6*). Although fluorescent probes have revealed the presence of Ca^2+^- and high pH vesicles in the foraminiferal cytoplasm during calcification (*7*, *8*), their transport and function remain a mystery. Therefore, understanding the foraminiferal calcification process would underpin the correspondence between organismal metabolic responses and global environmental changes within the marine carbon cycle.

**Fig. 1.**
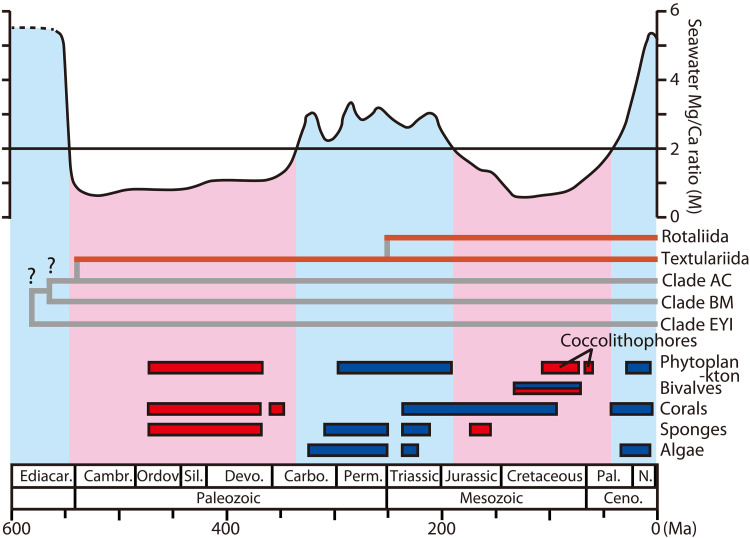
Seawater Mg/Ca ratio and fossil record. Geological fluctuations in the seawater magnesium/calcium (Mg/Ca) ratio (*14*), fossil records of major Foraminifera orders (*10*), and their phylogenetic relationships (*11*). Blue hatches (Mg/Ca > 2) indicate aragonite and high Mg calcite precipitation conditions, and pink hatches (Mg/Ca < 2) indicate low Mg calcite precipitation conditions in seawater at 25°C. Bars denote the accumulation of metazoans with aragonite and high Mg calcite (blue) and low Mg calcite (red). Clades EYI (Clade E: Psammophaga sp., Vellaria sp. + Clade Y: R. filosa, Cribrothalammina alba, and CladeY_allogromiid + Clade I: Allogromia sp., Astrammina triangularis, and Astrammina rara), BM (Bathysiphon argenteus and Micrometula sp.), AC (Clade A: Allogromiid + Clade C: Rhizammina algaeformis, Syringammina corbicula, Shinkaiya lindsayi) describe single-chambered cells with organic or agglutinated tests in class Monothalamea, Foraminifera (*11*). The divergence time of these clades is estimated to be in the Precambrian (*11*).

Foraminifera are the oldest known lineage of biomineral-producing eukaryotes (*9*). In the Cambrian, the earliest test-forming Foraminifera (order Textulariida, class Globothalamea) secreted CaCO_3_ to cement sand grains and form a test (Fig. 1) (*10*). The order Rotaliida, which is a robust monophyletic clade in Globothalamea, appeared in the Triassic as CaCO_3_ wall test-forming species (*11*). Throughout Earth’s history, the Mg/Ca ratio of seawater has fluctuated widely, principally because of the tectonically driven elevation of Ca^2+^ in seawater (i.e., ocean crust production) with atmospheric *p*CO_2_ induced by volcanic outgassing (*12*–*14*). This variability has prompted marine calcifying organisms (e.g., corals, bivalves, and sponges) to respond by precipitating either low Mg or high Mg CaCO_3_ (*14*). However, the order Rotaliida precipitated low Mg calcite in the Triassic when the seawater was under high Mg conditions. This order has continued to secrete low Mg CaCO_3_ throughout the last ~250 million years, even under the present high Mg seawater conditions (Fig. 1). Such geological and evolutionary records suggest that the calcification mechanism of Rotaliida is capable of functioning, despite large fluctuations in seawater chemistry over the Phanerozoic.

**Fig. 2.**
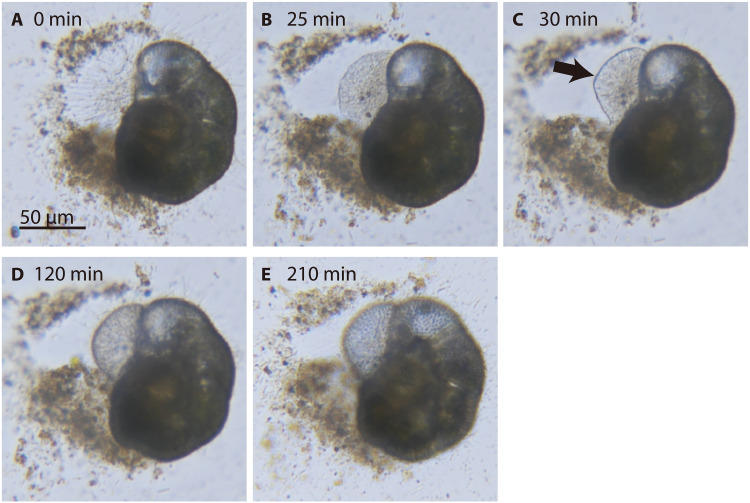
Calcification in Rotaliida, Foraminifera. (**A** to **E**) Chamber formation and calcification in *Ammonia beccarii*. (A) Fine reticulopodia extend from an aperture. (B) Dense filamentous reticulopodia define the new chamber. (C) A thin and fragile organic membrane (black arrow) is formed along the edge of the filamentous reticulopodia. (D) CaCO_3_ precipitates along the membrane. (E) The calcareous test thickens, and pores can be observed. Images of the chamber formation were acquired after 0 (A), 25 (B), 30 (C), 120 (D), and 210 (E) min.

Eukaryotic calcification is observed in many marine clades, and its primary function is to provide structural complexity, protection from predators, light refraction, and/or assistance during grazing (*15*). To date, sequence and genome analyses of multicellular mollusks, corals, and unicellular coccolithophores have revealed distinct calcification-related genes (*16*–*18*). However, it is unclear how Ca^2+^ and inorganic carbon (CO_2_ and bicarbonate: HCO_3_^−^) may be rerouted from other metabolic functions toward calcification, because carbon is an essential building block and energy source, whereas Ca^2+^ mediates intracellular signaling and controls numerous cellular processes (*19*, *20*). Rotaliida (the calcifying foraminifers) offer notable advantages in studying calcification, because they are amenable to metabolic regulation analysis via single-cell RNA sequencing (RNA-seq) and grow by sequentially adding calcareous chambers to their tests (Fig. 2).

Here, we hypothesized that the calcification mechanism in Rotaliida involves specific control of intracellular and extracellular Ca^2+^ levels. The genus *Ammonia* is one of the well-studied Rotaliida to understand cellular processes resulting from foraminiferal calcification, because these foraminifers can be maintained under laboratory conditions and their calcification processes can be precisely observed via microscopic observation (*21*–*23*). Therefore, we extracted, sequenced, and compared mRNA from single cells during the calcification and noncalcifying stages to identify calcification-related genes. Using comparative transcriptomics, we predicted the molecular mechanism of calcification developed by this unicellular eukaryote and suggested the evolution of foraminiferal calcification through geological changes. Given the critical role of Rotaliida in the evolution of biomineralization, these organisms may prove to be the best geochemical indicators for predicting upcoming global changes.

## RESULTS

### Cellular-level changes during chamber formation

In the present study, the calcifying Rotaliida species *Ammonia beccarii* was observed forming a new calcareous chamber over 6 to 8 hours every 1 to 2 days under laboratory conditions (Fig. 2). Reticulopodia, common pseudopodia in Foraminifera, are usually extended in all directions from the aperture of the test. At the onset of chamber formation, reticulopodia were gathered on the side of the aperture to regulate the shape and space of a new chamber and make dense filamentous meshwork (Fig. 2, A and B). These reticulopodia are composed of finger-like protuberances and a dense meshwork of F-actin supported by microtubules (*24*). Along the edge of the filamentous reticulopodia, an organic membrane was formed (Fig. 2C). CaCO_3_ was precipitated along this membrane and formed a thick CaCO_3_ test (Fig. 2, D and E). This new chamber was sequentially filled by cytoplasm. This result shows that foraminifers certainly calcified during chamber formation.

**Fig. 3.**
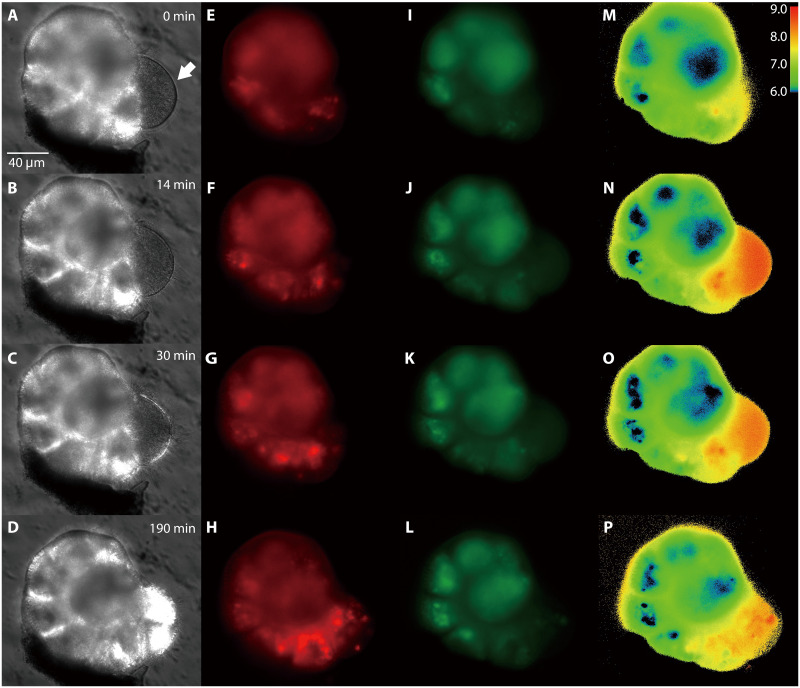
Time series showing simultaneous images of Ca^2+^ and pH during calcification in *A. beccarii*. (**A** to **D**) Differential interference contrast images during calcification. The white arrow indicates the position of a new chamber. (**E** to **H**) Intracellular Ca^2+^ images with Rhod-3. Bright red spots show Ca^2+^ vesicles. (**I** to **L**) Intracellular pH images with pyranine. Bright green spots show H^+^ vesicles. (**M** to **P**) Intracellular pH gradient. High pH is indicated by orange-red color. Images acquired after 0 [the first line: (A), (E), (I), and (M)], 14 (B, F, J, and N), 30 (C, G, K, and O), and 190 (D, H, L, and P) min.

The intracellular states during chamber formation were observed via simultaneous imaging (Fig. 3). Differential interference contrast images emphasized the filamentous meshwork of reticulopodia and the organic membrane (Fig. 3, A and B). CaCO_3_ precipitation began on the organic membrane, and subsequently the CaCO_3_ layer thickened (Fig. 3, C and D). The calcium indicator Rhod-3 AM images indicated a high concentration of Ca^2+^ in the cytoplasm, particularly in the three young chambers as well as the presence of Ca^2+^ vesicles (Fig. 3, E to G). These Ca^2+^ vesicles were transferred to the site of a new chamber, as seen in the time series (Fig. 3H and movie S1). Similarly, the pH images showed low pH (~6 to 8) vesicles in the three young chambers (Fig. 3, I to L). In contrast to the presence of low pH vesicles, the pH gradient images indicated high pH (~8 to 9) in the cytoplasm around the new chamber (Fig. 3, M to P); the highest pH (~9) was observed at the beginning of CaCO_3_ precipitation (Fig. 3N). These intracellular images showed that the distribution and transport of Ca^2+^ vesicles associated with low pH vesicles in high pH cytoplasm around the calcification site (new chamber).

### Discovery of calcification-related genes in the Rotaliida foraminifer

**Fig. 4.**
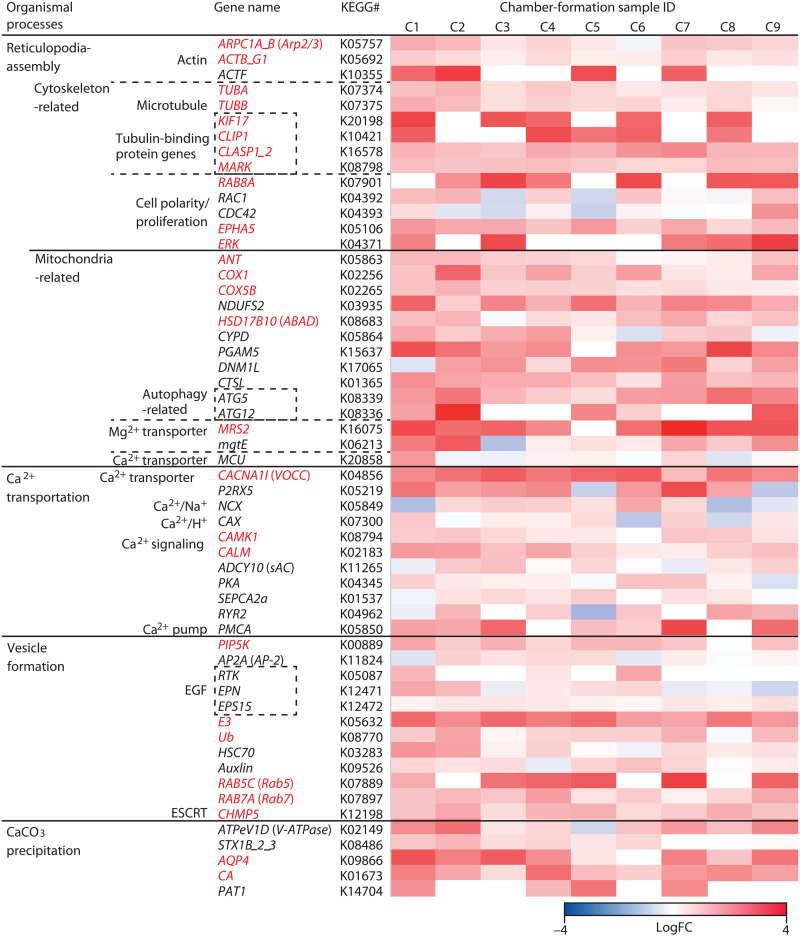
List of 56 genes highly expressed during calcification and their expression level. The expression level is shown by log fold change (FC) between each calcification condition (C1 to C9) and an average of control (N1 to N3) conditions. The genes, which are significantly expressed during calcification (*P* < 0.05), are indicated by red color. KEGG, Kyoto Encyclopedia of Genes and Genomes; Arp2/3, actin-related proteins 2/3 (ARPC1A_B); ACTF, actin; ACTB_G1; TUBA/TUBB, tubulin-α/β; RAB8A and RAC1, Ras-related proteins; ERK, mitogen-activated protein kinase 1/3; EPHA5, Eph receptor A5; ANT, adenine nucleotide translocase; ABAD, amyloid-binding alcohol dehydrogenase; CYPD, cyclophilin D; PGAM5, serine/threonine-protein phosphatase; DNM1L, dynamin-related guanosine triphosphate (GTP)ase; ATGs, autophagy-related genes; VOCC, voltage-dependent calcium channels; CALM, calmodulin; CAMK, calcium/CALM-dependent protein kinase; PIP5K, 1-phosphatidylinositol-4-phosphate 5-kinase; E3, ubiquitin ligase; Ub, ubiquitin C; HSC70, heat shock 70 kDa protein 1/8; CA, carbonic anhydrase; AQP4, aquaporin 4; PAT1, putative anion transporter; COX1 and COX5B, cytochrome c oxidase subunits I and V.

To identify the molecular mechanisms responsible for calcification in *A. beccarii*, we sequenced mRNAs from nine single-cell samples (C1 to C9) isolated during chamber formation (calcification stage) and three samples (N1 to N3) isolated during the intermediate life stage (noncalcifying control). A reference gene expression profile was obtained on the basis of a mixture of 100 specimens (R100) collected at various cell stages (see Materials and Methods and table S1). From R100 and C1 reads, 54,914 scaffolds were assembled de novo, and 48,542 coding sequences (CDSs) longer than 80 amino acids were selected for RNA-Seq analyses. The reads of the 12 single-cell samples were separately mapped to the CDSs; reads per kilobase per million (RPKM) mapped reads were calculated for calcifying versus control conditions. The gene expression profiles of C1 to C9 samples differed from those of N1 to N3 samples (fig. S1). Specifically, 7390 CDSs were significantly expressed (*P* < 0.05, DESeq2 analysis) or were only expressed under calcification conditions (see Materials and Methods and fig. S2). On the basis of these highly expressed genes, 56 candidates (27 with *P* < 0.05 and 29 with *P* > 0.05) were assessed to estimate the metabolic pathways corresponding to the cellular processes involved in reticulopodia assembly, Ca^2+^ transport, and CaCO_3_ precipitation (Figs. 2 and 4 and table S2).

### Candidate genes controlling foraminiferal calcification

**Fig. 5.**
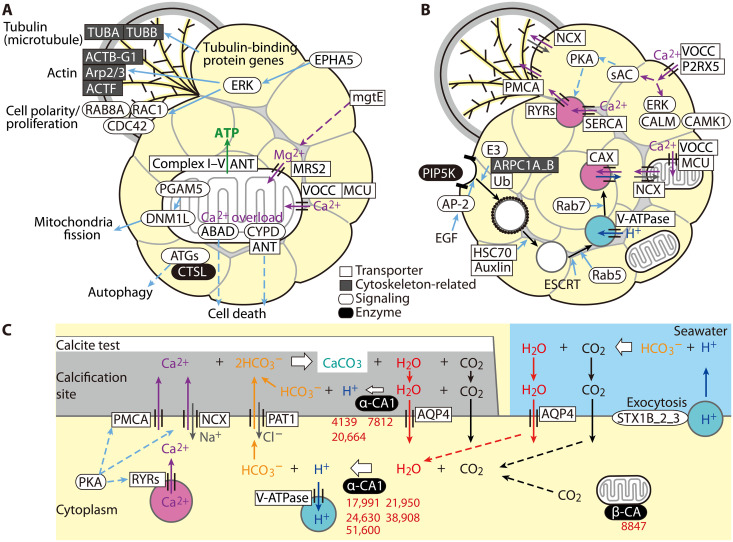
Predicted metabolic pathways and transport associated with foraminiferal calcification. (**A** to **C**) Schematic view of foraminiferal calcification. (A) Highly expressed genes related to the cytoskeleton (microtubules, actin, and cell polarity/proliferation) and mitochondria (Ca^2+^ and Mg^2+^ channels, mitochondrial damage, mitochondrial fission, and autophagy). (B) Highly expressed genes related to Ca^2+^ transport: Ca^2+^ channels activated at plasma and mitochondrial membranes, Ca^2+^ signaling, Ca^2+^ channels and pumps, as well as vesicle endocytosis and clathrin coating for vesicles that store excess Ca^2+^ released from the mitochondria. (C) CaCO_3_ precipitation at a new chamber site based on Ca^2+^ transport and HCO_3_^−^ generation from H_2_O and CO_2_ by α-CA. The numbers in red denote contigs encoding α-CA or β-CA.

Cytoskeleton-related genes (i.e., actin and tubulin) and cell polarity– and proliferation-related genes were significantly or highly expressed during chamber formation (Fig. 4). Actin-related proteins 2/3 (Arp2/3) complex (ARPC1A_B), actin (ACTF), and actin beta/gamma 1 (ACTB_G1) are generally required for the assembly of adherence junctions and the regulation of the actin cytoskeleton in eukaryote (Fig. 5A) (*25*). Tubulin-α (TUBA) and tubulin-β (TUBB) are related to microtubule polymerization (*26*) together with tubulin-binding proteins (*27*–*30*). We detected strong expression of the genes related to Ras-related proteins (RAB8A and RAC1) and cell division control proteins (CDC42) as well as mitogen-activated protein kinase 1/3 (ERK) and Eph receptor A5 (EPHA5), determining cell polarity, proliferation, size, and shape (*31*–*35*). These findings indicate the development of the cytoskeleton and cell polarity consistent with cell observation (Fig. 2, A and B). At the same time, mitochondria-related genes were highly expressed (Fig. 4). Cytochrome c oxidase subunits I and V (COX1 and COX5B) are associated with respiratory H^+^ pumps (complexes I to IV) across the inner mitochondrial membrane (*36*), and nicotinamide adenine dinucleotide dehydrogenase participated in mitochondrial oxidative phosphorylation to support mitochondrial adenosine triphosphate (ATP) synthesis (Fig. 5A). Accordingly, adenine nucleotide translocase (*ANT*) was significantly expressed to transport ATP from the mitochondrial matrix to the cytoplasm. These up-regulations suggest active mitochondrial ATP synthesis during chamber formation, because foraminiferal cells could consume abundant energy for reticulopodia assembly and increase cell volume. In contrast, significant expression of amyloid-binding alcohol dehydrogenase (*ABAD*) was identified with high expression of cyclophilin D (*CYPD*) (Fig. 4). These genes are directly related to mitochondrial toxicity, which damages mitochondria, leading to cell death (Fig. 5A) (*37*). However, serine/threonine-protein phosphatase (*PGAM5*), dynamin-related guanosine triphosphate (GTP)ase (*DNM1L*), and autophagy-related genes (ATGs) were also highly expressed (Fig. 4). These gene expressions related to mitochondrial fission and autophagy suggest that the number of mitochondria is maintained and that the damaged mitochondria are recycled via autophagy.

The genes associated with the transportations of Ca^2+^ and Mg^2+^ showed opposite expression trends. Only two genes, mitochondrial magnesium transporter (*MRS2*) and plasma membrane magnesium transporter (*mgtE*), showed high expression levels (Figs. 4 and 5A); MRS2 stimulates Mg^2+^-dependent enzyme to accelerate ATP synthesis in mitochondria (*38*). Any significant changes in expressions of genes related to the excretion of Mg^2+^ across the plasma membrane were not detected. In contrast, many calcium and Ca^2+^-related genes showed high expression during chamber formation (Fig. 4). Significant expression of voltage-dependent calcium channels (VOCC) showed active Ca^2+^ uptake from ambient seawater to cytoplasm and transport from cytoplasm to mitochondria (Fig. 5B). Mitochondrial Ca^2+^ plays a primary role in activating the tricarboxylic acid cycle through allosteric regulation of some enzymes and in stimulating ATP synthase and ANT during oxidative phosphorylation (*39*). Cytosolic Ca^2+^ stimulated various signaling pathways and metabolic processes (i.e., proliferation) (*19*) as shown by the significant expression of calmodulin (*CALM*) and calcium/CALM-dependent protein kinase (*CAMK*) (Figs. 4 and 5B). Moreover, high expression levels of genes related to vesicle generation were detected (Fig. 4). Significant expression of 1-phosphatidylinositol-4-phosphate 5-kinase (*PIP5K*), ubiquitin ligase (*E3*), and ubiquitin C (*Ub*) indicated clathrin-dependent endocytosis at the onset of vesicle formation (Fig. 5B) (*40*). Such clathrin-coated vesicles are uncoated by heat shock 70-kDa protein 1/8 (HSC70) and auxlin and transported to endosomes for lysosome biogenesis via activation of Rab GTPase (RAB5 and RAB7) (*41*, *42*). Furthermore, sarco/endoplasmic reticulum Ca^2+^–adenosine triphosphatase (ATPase) (SERCA) and ryanodine receptors (RYRs) showed high expression (Fig. 4). These Ca^2+^ pumps located on vesicle membranes may transport and export free Ca^2+^ between the cytosol and vesicles (*19*, *43*). High expression of calcium/proton antiporter (*CAX*) could indicate the uptake of Ca^2+^ into vesicles. These gene expressions suggest the formation of Ca^2+^ vesicles. One of our important findings was the high expression of P-type Ca^2+^ transporter type 2B (*PMCA*) with the stimulation of protein kinase A (*PKA*) and soluble adenylate cyclase (*sAC*) (Figs. 4 and 5B). This membrane transporter is involved in Ca^2+^ excretion outside the cytoplasm (*19*).

Another crucial finding was the significant expression of carbonic anhydrase (*CA*) and aquaporin 4 (*AQP4*) (Fig. 4). The enzyme CA (EC4.2.1.1) catalyzes the interconversion of H_2_O + CO_2_ to HCO_3_^−^ + H^+^ (*44*). The integral membrane protein AQP4 allows the passage of water molecules through the cell membrane and supplies H_2_O for further HCO_3_^−^ production around the cell membrane (*45*). CO_2_ can be diffused from seawater as well as calcification sites and produced during metabolic processes (e.g., the tricarboxylic acid cycle). These *CA* and *AQP4* expressions imply the intracellular generation of HCO_3_^−^ (Fig. 5C). Moreover, vacuolar-type ATPase (*V-ATPase*), which acts as a proton pump on biological membranes, was also highly expressed during calcification (Fig. 4). High expression of the exocytosis-related protein syntaxin 1B/2/3 (*STX1B_2_3*) indicates that such H^+^ vesicles are likely released via exocytosis (*46*). The intracellular HCO_3_^−^ is released to the calcification site via the putative anion transporter (PAT1), which exchanges intracellular HCO_3_^−^ and extracellular chlorine ion (Cl^−^).

### Diversity of calcium-related genes

**Fig. 6.**
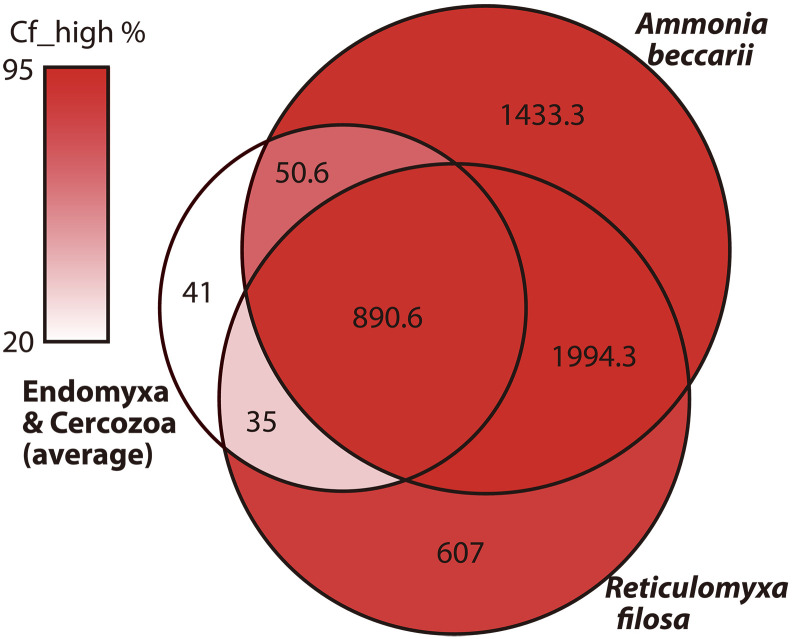
Venn diagram of calcium-related proteins. Number of protein motifs belonging to calcium-related proteins in test-forming (*A. beccarii*) and naked (*R. filosa*) Foraminifera as well as in three endomyxan and cercozoan species. Circle size indicates the number of protein motifs, and color denotes the relative abundance of protein motifs with high expression genes during calcification (Cf).

To characterize the active uptake and transport of Ca^2+^, we used the Pfam database (*47*) and identified 4369 open reading frames (ORFs) representing 245 calcium-related motifs in the Rhizaria supergroup, including the naked foraminifer *Reticulomyxa filosa* (basal foraminiferal clade EYI in Fig. 1) (*11*), two members of subphylum Endomyxa, and phylum Cercozoa (see Materials and Methods and table S3). The number of ORFs annotated as calcium-related motifs was fourfold greater in *A. beccarii* than the average number for Endomyxa and Cercozoa (~1018 ORFs) and ~1.3 times greater than that for the naked foraminifer, *R. filosa* (~3527 ORFs) (Fig. 6). Rhizaria shared ~890 ORFs corresponding to calcium-related genes, whereas foraminifers (*A. beccarii* and *R. filosa*) shared ~1994 ORFs. The high diversity of ORFs in the basal foraminifer *R. filosa* suggests that a common ancestor of Foraminifera had several calcium-related genes. Moreover, test-forming *A. beccarii* presented ~1433 ORFs that were absent from *R. filosa* or other rhizarians (Fig. 6) and were primarily annotated as motifs of calcium-binding (e.g., EF-hand) and calcium-signaling (G alpha subunit) proteins as well as calcium channel, sodium/calcium antiporter motifs (VGCC_alpha and Calx_beta), and calcium-dependent proteins (table S3). Almost all calcium-related genes were highly expressed during calcification (Fig. 6). In particular, the wide array of calcium-binding proteins, VGCC_alpha and Calx_beta, was congruent with the up-regulation of calcium and Ca^2+^-related genes.

### Evolution of foraminiferal CA

In the present study, *CA* genes were identified in Foraminifera for the first time. *CA*s are encoded by five nonhomologous gene families (α to ζ) that evolved independently in prokaryotes and eukaryotes (*48*–*50*). We conducted phylogenetic analyses comparing *CA*s from *A. beccarii*, another calcite test-forming foraminifer (Rotaliida) *Globobulimina* sp., the naked foraminifer *R. filosa*, two Endomyxa, and one Cercozoa (see Materials and Methods and fig. S3). The two Rotaliida (test-forming foraminifers) had both α- and β-CA members, whereas the naked foraminifer had only β-CAs, and other Rhizaria taxa had only α-CAs (fig. S4).

**Fig. 7.**
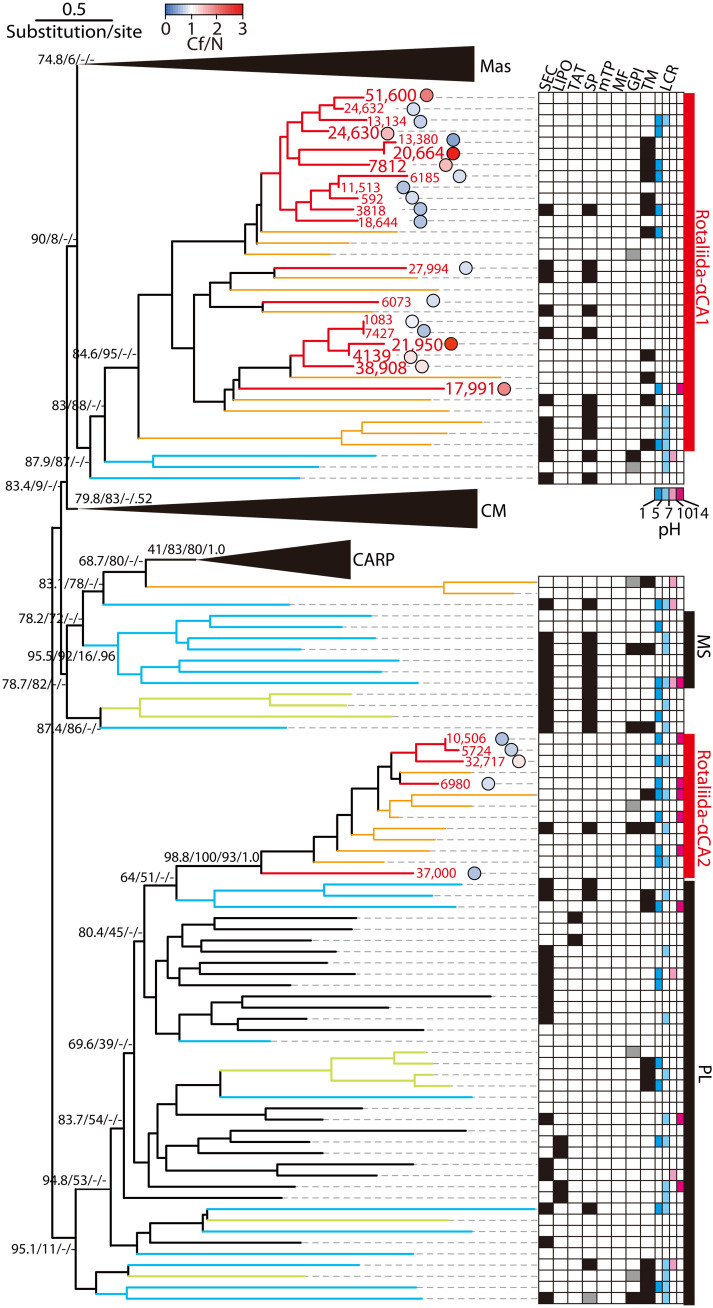
Phylogeny of α-CA protein sequences. Rotaliida-α-CA1 and Rotaliida-α-CA2 are shown along the membrane-associated/secreted (Mas), cytosolic/mitochondrial (CM), CA-related protein (CARP), molluscan-specific (MS), and prokaryotic-like (PL) clades (*51*, *52*). Line colors indicate taxa: *A. beccarii* (red), *Globobulimina* sp. (orange), the cercozoan *B. natans* and endomyxans *S. subterranea* and *P. brassicae* (light green), other eukaryotes (light blue), and Bacteria (black). The expression ratio of each *A. beccarii* contig between calcification (Cf) and control conditions (N) is shown in blue-red colors. Each expression ratio of the open reading frame (ORF) is shown by the circle with this color legend. In silico prediction of specific motifs and domains: secretory signal peptide (SEC), lipoprotein signal peptide (LIPO), twin-arginine translocation signal peptide (TAT), signal peptide (SP), mitochondria transit peptide (mTP), mitochondria transit peptide predicted by MitoFates (MF), glycosylphosphatidylinositol anchor signal (GPI), and transmembrane (TM) (see Materials and Methods for details). Low-complexity regions (LCRs) and their theoretical pH are shown.

Among five CA families, α-CAs are involved in CO_2_/HCO_3_^−^ interconversion and calcification in mollusks, sea urchins, and corals (*48*–*50*). Phylogenetic analysis was conducted, including representative α-CA sequences categorized as membrane-associated/secreted, cytosolic/mitochondrial, and CA-related proteins (table S4) (*51*, *52*). The α-CA protein sequences of two test-forming foraminifers formed two clades: Rotaliida-α-CA1 and Rotaliida-α-CA2 (Fig. 7 and fig. S4). The sequences of *Globobulimina* sp., which were of these two clades, could be unambiguously obtained in *Globobulimina* sp. Rotaliida-α-CA1 formed an independent clade from metazoans, whereas Rotaliida-α-CA2 clustered in the prokaryotic-like clade. Some Rotaliida-α-CA1 (ORFs 51,600, 20,664, and 21,950) were expressed only or more strongly during calcification (Fig. 7), and some (ORFs 4139, 7812, 20,664, and 24,630) were predicted to be secreted/membrane-bound proteins with acidic low-complexity regions (LCRs) (Fig. 7 and tables S4 to S9). In mollusks, calcification-related α-CAs contain acidic LCRs, which could bind Ca^2+^ leading to CaCO_3_ nucleation (*50*). Moreover, our in silico protein motif predictions indicated that Rotaliida-α-CA1 comprised both secretory α-CAs (ORF 7812) and intracellular α-CAs (ORF 21,950) (Fig. 5C). The former are likely involved in HCO_3_^−^ generation and calcium binding at the calcification site. The latter do not interact with Ca^2+^, as they lack acidic LCRs; hence, they likely participate in HCO_3_^−^ production in the cytosol.

**Fig. 8.**
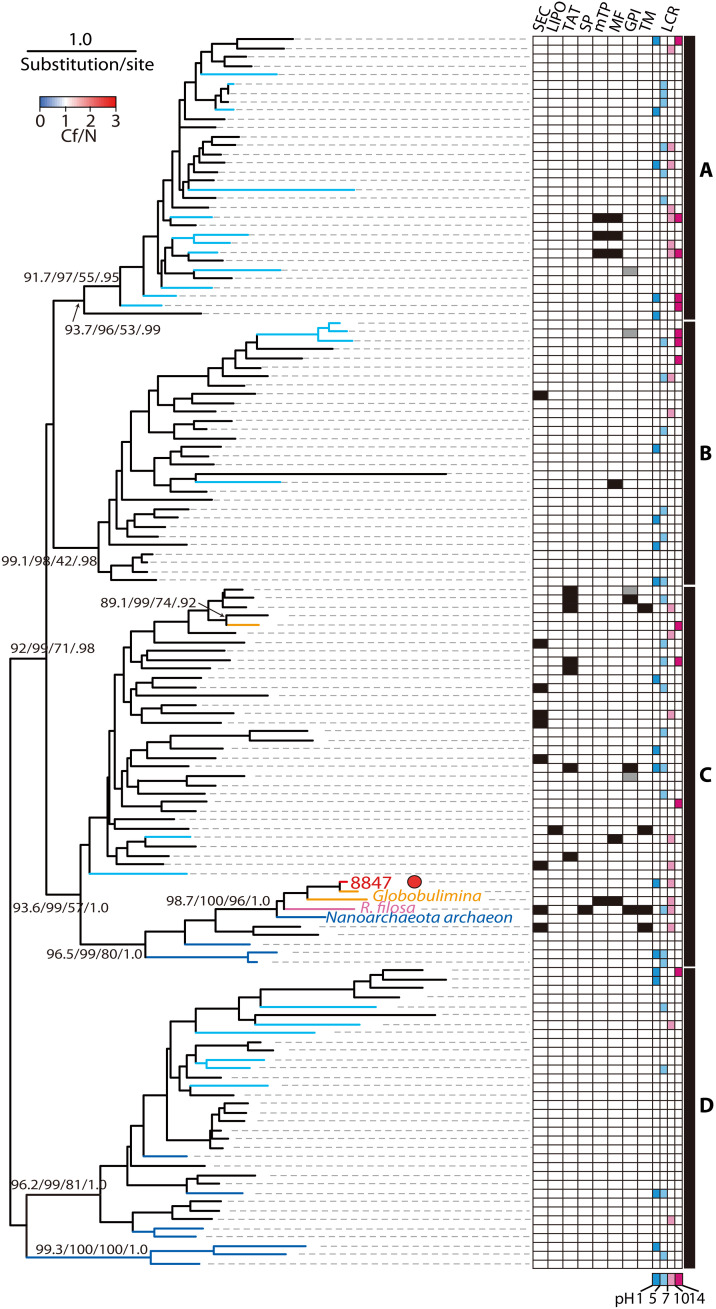
Phylogeny of β-CA protein sequences. The monophyletic clade of Foraminifera is placed in clade C. Line colors include Archaea (blue) in addition to other taxa as in Fig. 7. In silico prediction of specific motifs and domains and the theoretical pH of LCRs are the same as those in Fig. 7.

β-CA of *A. beccarii* showed 2.7 times higher expression during calcification than under control conditions. β-CA members are widely found in Bacteria, Archaea, and the chloroplasts of plants (*53*, *54*) and have been reported in invertebrates and protists (*54*, *55*). On the basis of phylogeny, eukaryotic β-CAs are sporadically placed in each of the four clades (A to D) as well as a sequence of *Globobulimina* sp., except for the chloroplast monophyletic clade (Fig. 8 and fig. S5) (*53*, *54*). β-CA protein sequences of three foraminiferal species (*A. beccarii*, *Globobulimina* sp., and *R. filosa*) formed a monophyletic clade. Although the N terminus of the *R. filosa* sequence was truncated, the sequences of all three were conserved among them, regardless of the test-forming ability. The foraminiferal clade is a sister to *Nanoarchaeota archaeon*, a distant lineage from the Asgard Archaea (*56*), suggesting that foraminiferal β-CA is derived by stepwise horizontal gene transfer from Archaea.

## DISCUSSION

Comparative transcriptomic analysis successfully extracted candidate genes that could play essential roles in the calcification of the rotaliid foraminifer *A. beccarii* (Fig. 4 and table S2). These gene expressions, in conjunction with the physiological images recorded, allow us to predict the metabolic pathways that drive the molecular mechanisms of foraminiferal calcification (Fig. 5). Foraminiferal calcification differs from that in other organisms, such as mollusks, corals, and coccolithophores, in which it is closely linked to chamber formation. In this process, a dense meshwork composed of reticulopodia very specifically regulates the shape and volume of a new chamber (Fig. 2, A and B) (*21*, *24*), as evidenced by the elevated expression of cytoskeleton-related genes (i.e., actin and tubulin) and cell polarity– and proliferation-related genes (Figs. 4 and 5A). Moreover, Ca^2+^ concentration was increased in the cytoplasm (as shown by the Rhod-3 AM image in Fig. 3 (E to H) along with the active uptake of Ca^2+^ from ambient seawater through ion channels VOCC and mitochondrial calcium uniporter (MCU) (Fig. 4). Cytoplasmic Ca^2+^ stimulates calcium signaling, involving CALM and CAMK, which are associated with intracellular movements as one of their multiple functions (*19*). Our prediction in Pfam accordingly suggested that *A. beccarii* has EF-hand proteins, which are related to such intracellular movements, as well as other calcium-related genes with high expression (Fig. 6 and table S3). These findings indicate that this foraminifer uses Ca^2+^ in the arrangement of reticulopodia for chamber formation. Elevated Ca^2+^ in the mitochondrial matrix up-regulates ATP synthesis and ANT to provide sufficient energy for chamber formation; the process involves significant expression of *COX1* and *COX5B* (*36*, *39*). However, excessive Ca^2+^ in the mitochondria is known to damage their membranes, which causes the collapse of mitochondrial membrane potential, ATP depletion, and rapid progression to cell death (e.g., apoptosis) (*37*), as suggested by the high expression of *ABAD*, *CYPD*, and *ANT* (Figs. 4 and 5A). Damaged mitochondria were presumably recycled via autophagy, and their numbers were maintained by mitochondrial fission, as illustrated by high expressions of *PGAM5*, *DNM1L*, and *ATGs*. Mitochondria likely maintain a constant energy supply for chamber formation. Thus, although Ca^2+^ promotes ATP synthesis for chamber formation, it also promotes cell death.

Numerous cellular processes are regulated by Ca^2+^, and at the same time the intracellular concentrations of Ca^2+^ are maintained at generally low levels (~100 nM in resting conditions) to avoid cell death (*19*). Because chamber formation in *A. beccarii* involves active uptake of Ca^2+^, their intracellular Ca^2+^ concentrations must be tightly controlled. To maintain optimal intracellular Ca^2+^ concentrations, the endoplasmic reticulum and the vesicles act as storage organs for calcium compounds in eukaryotic cells (*19*, *43*). During foraminiferal calcification, the vesicles are generated through clathrin-dependent endocytosis to lysosome biogenesis, as suggested by the high expression of the related genes: *PIP5K*, *E3*, *Ub*, *HSC70*, *Auxilin*, and *Rab GTPase* (Figs. 4 and 5B) (*40*–*42*). Ca^2+^ pumps on vesicle membranes may transport free Ca^2+^ in the cytosol into the vesicles (*19*, *43*), as indicated by the high expressions of *SERCA* and *RYRs* (Figs. 4 and 5B). Rhod-3 AM images (Fig. 3, E to H, and movie S1) also demonstrated Ca^2+^-storing vesicles and their transportation toward the calcification site; excess Ca^2+^ was released from the mitochondria and stored in vesicles. This may be a dual mechanism of reducing Ca^2+^-induced cellular damage associated with energy production and supplying Ca^2+^ for calcification. Subsequently, Ca^2+^ was pumped out via membrane transporters, including PMCA and solute carrier family 8 (NCX), under the stimulation of PKA and sAC, toward the calcification site (Fig. 5, B and C). These results suggest a well-coordinated Ca^2+^ trafficking system.

Facilitation of Ca^2+^ utilization in foraminifers is also suggested by the high diversity of calcium-related motifs, which were annotated on the basis of the Pfam database (Fig. 6 and table S9). Both naked and test-forming foraminifers have a far greater number of calcium-related genes than other rhizarians. Foraminifera are generally giants among unicellular eukaryotes, ranging from tens to hundreds of micrometers in cell size (*57*), and have numerous mitochondria in a cell (*58*). To manage cellular processes in such a giant cell, foraminifers could require mitochondrial ATP synthesis activated by Ca^2+^ usage. Test-forming *A. beccarii* showed rich variation in calcium-related genes, which were highly expressed during calcification (Fig. 6). These annotated genes (i.e., calcium-binding and calcium-signaling proteins, Ca^2+^ channels) are precursors for calcification, associated with the core calcification pathway predicted in the present study (Fig. 5C). Therefore, Ca^2+^ vesicles could serve as stock or waste, whereas Ca^2+^ transporters could help regulate the intracellular concentration of Ca^2+^ in *A. beccarii*.

Calcite tests of such Rotaliida species contain very little Mg [~250 parts per million (ppm)] or two orders of magnitude less than that in abiotic precipitated CaCO_3_ (~100,000 ppm) in seawater (*6*); this difference may be attributed to the active excretion of Mg (*6*, *8*). However, the transcriptomic analyses indicated no significant expression of Mg^2+^-related genes except for the high expression of *MRS2*, which enables Mg^2+^-dependent mitochondrial ATP synthesis (Figs. 4 and 5A) (*38*). This result refutes the active uptake and excretion of Mg^2+^ hypothesized in the previous studies (*6*, *8*). Instead, calcium-related genes are likely the main determinants of Mg/Ca composition in Rotaliida calcite. The active usage of Ca^2+^ with Ca^2+^ trafficking system during calcification could result in precipitation of low Mg CaCO_3_ in Rotaliida species regardless of ambient environmental Mg/Ca ratio.

Significant expression of *CA* indicated HCO_3_^−^ supply for calcification in *A. beccarii* via the interconversion of H_2_O and CO_2_ (Figs. 4 and 5C). Both naked and test-forming foraminifers have β-CA (of the five nonhomologous gene families, α to ζ) (Fig. 8). The physiological role of β-CA is poorly understood, although assays in bacteria indicate that it provides HCO_3_^−^ for oxaloacetate synthesis (*59*). In eukaryotes, both in silico predictions and experiments in fungi and fruit flies have shown that β-CAs localize to mitochondria (*55*, *60*). Our in silico predictions also showed that the β-CA sequence in *Globobulimina* sp. was localized to mitochondria, suggesting that the same may apply to other foraminiferal β-CAs given their conserved sequences. Oxaloacetate is generally associated with mitochondrial ATP synthesis as an intermediate in the tricarboxylic acid cycle. In *A. beccarii*, β-CA likely contributes to mitochondrial ATP synthesis, as its high expression was observed in the present study.

Test-forming Rotaliida species also had α-CA (Fig. 4 and fig. S3). Although the α-CA family comprises numerous isoforms with different enzymatic activities (e.g., photosynthesis), kinetic properties, sensitivity to inhibitors, and subcellular localization (*50*), *A. beccarii* does not have photosynthetic symbionts. Instead, the secreted/membrane-bound α-CA with acidic LCRs likely acts as a Ca^2+^-binding factor for CaCO_3_ nucleation as shown in mollusks (*50*). The phylogenetic analysis of α-CAs with the prediction of their signal peptides in addition to significant expressions of CA genes indicated that Rotaliida–αCA1 likely catalyzes CO_2_/HCO_3_^−^ interconversion, which is related to foraminiferal calcification. The generated HCO_3_^−^ are supplied to the calcification site via the anion transporter (PAT1) (Fig. 5C). Moreover, we found that Rotaliida–α-CA1 showed two different distributions: at the extracellular site and in the cytosol (Fig. 5C). This strategy enables the utilization of CO_2_ derived from both extracellular and intracellular origins. Congruently, the pH gradient images showed high pH, which could be attributed to HCO_3_^−^, in the cytoplasm around the new chamber (Fig. 3, N to P). The presence of low pH vesicles suggested that H^+^ was released into the cytosol upon catalysis by Rotaliida–αCA1 and sequentially recruited into H^+^ vesicles through V-ATPase (Figs. 4 and 5C). In our prediction, these H^+^ vesicles could be released via exocytosis, lowering the pH of ambient seawater. A previous fluorescent microscopic study, which focused on the pH changes of ambient seawater during foraminiferal calcification, reported a lowering of pH (~6.9) due to H^+^ release and decomposition of HCO_3_^−^ to generate CO_2_ and H_2_O (*21*). AQP4 found in our transcriptomic analysis could transport H_2_O molecules (Fig. 5C). Thus, the HCO_3_^−^ used during foraminiferal calcification originates from multiple sources of CO_2_ and H_2_O. This likely explains that multiple sources, particularly those of CO_2_, serve as a physiological control mechanism for δ^18^O and δ^13^C in foraminiferal tests via HCO_3_^−^ production. Such physiology could be partly responsible for the nonequilibrium state of δ^18^O and δ^13^C between foraminiferal tests and the environment observed in geochemical studies (*3*).

As observed for *A. beccarii*, the Rotaliida species precipitate low Mg CaCO_3_ regardless of the environmental conditions (Fig. 1). The development of calcium-related genes in *A. beccarii* implies that the active utilization of Ca^2+^ may cause predominance of calcium rather than magnesium during CaCO_3_ precipitation. However, the Mg/Ca ratio in seawater has most likely dictated biotic calcification with low or high Mg throughout Earth’s history (*12*–*14*). At the Permian/Triassic boundary, a large amount of CO_2_ was released into the atmosphere due to continental eruptions, resulting in strong warming and ocean acidification (*12*, *13*). Although high Mg CaCO_3_ tends to dissolve during ocean acidification (*14*), low Mg CaCO_3_ precipitated by Rotaliida might be more resistant to dissolution. Moreover, calcification in *A. beccarii* lowers the pH of ambient seawater by releasing H^+^ for CO_2_/HCO_3_^−^ interconversion via Rotaliida-αCA1 (Figs. 3, I to L, and 5C). The pH of ambient seawater during calcification is 6.9 (*21*), which is considerably lower than the typical seawater pH of 7.8 to 8.4 (*1*), suggesting that Rotaliida are capable of calcifying under acidic conditions. Because foraminifers reuse CO_2_ via H^+^ generation (Fig. 5C), they may use both CO_2_ and HCO_3_^−^, which are highly soluble under acidic conditions. Thus, the unique evolution of Rotaliida calcification might work favorably against environmental change in geological history. Future studies are warranted to address the changes in *CA* expression corresponding to low pH seawater over time.

In summary, the present study successfully reconstructs the metabolic pathway driving calcification in a unicellular eukaryote. Regardless of calcareous test formation, foraminifers predominantly use Ca^2+^ and β-CA to facilitate mitochondrial ATP production, likely enabling the growth of such large cells. At the same time, to avoid cell death, the intracellular concentration of Ca^2+^ is controlled by the removal or storage of excess Ca^2+^ in vesicles (Fig. 5, A and B). Contrary to expectations (*6*), Rotaliida species do not actively take up or excrete Mg^2+^ during calcification. Along with the evolution of Ca^2+^ utilization, the role of α-CA was established after the early diversification of foraminiferal lineages. Rotaliida-αCA1 facilitates HCO_3_^−^ generation, resulting in CaCO_3_ secretion using Ca^2+^ excreted from the cell. Given the independent evolution of both Ca^2+^-related and CA genes, foraminiferal calcification appears essential for sustaining the growth of outsized cells. Such unique evolution could have enabled Rotaliida to survive global fluctuations in seawater Ca^2+^ and CO_2_, and it may confer an advantage to them in the wake of global changes.

## MATERIALS AND METHODS

### Sample collection and culture

Surface sediments (top: 5 mm) were collected from the brackish water salt marsh of Hirakata Bay, Natsushima-cho Yokosuka, Japan (35°19′21′′N, 139°38′5′′E). Living specimens of *A. beccarii* were collected from the sediment samples and cleaned in 0.2-μm membrane-filtered seawater under a microscope. Five specimens were placed in a petri dish with a culture medium containing a 1× penicillin-streptomycin-neomycin mixture (Thermo Fisher Scientific, Waltham, MA, USA) in 0.2-μm membrane-filtered seawater. After incubation at 20°C for 3 to 7 days, the medium was replaced with 0.2-μm filtered seawater, and the specimens were cultured for a few more weeks. The foraminifers were fed weekly with dried microalgae (*Dunaliella tertiolecta*, NIES-2258) previously frozen at −30°C for half a year to prevent contamination with their RNA. Some foraminifers started growing and formed chambers within 1 to 2 days after feeding. On the basis of microscopic observations, we selected nine specimens (i.e., cells) at different stages of chamber formation (C1 to C9) as well as three specimens from the intermediate life stage (N1 to N3). The reference R100 specimens were collected at various cell stages. Each single-cell sample was preserved in a microtube and stored in liquid nitrogen. R100 specimens were preserved in NAsafe solution [4 M (NH_4_)_2_SO_4_, 10 mM EDTA, 0.1 M 2-(N-morpholino)ethanesulfonic acid (pH 4.6)] and stored at 4°C.

### RNA extraction

Total RNA was extracted from the R100 sample and purified using the SV Total RNA Isolation System (Promega, Madison, WI, USA). After removing the NAsafe solution, all specimens were crushed in liquid nitrogen with an SK mill (Tokken Inc., Chiba, Japan) and mixed with SV RNA lysis buffer. RNA extraction and purification were conducted following the manufacturer’s instructions. RNA quality and quantity were evaluated using an Agilent High Sensitivity DNA assay on a Bioanalyzer 2100 (Agilent Technologies, Santa Clara, CA, USA).

### RNA-seq library preparation and transcriptome sequencing

Purified RNA from the R100 sample was reverse-transcribed into cDNA with oligo(dT) primer and amplified for 13 cycles using the SMART-Seq v4 Ultra Low Input RNA kit (Clontech Laboratories Inc., Mountain View, CA, USA). Single-cell samples (C1 to C9 and N1 to N3) were directly reverse-transcribed into cDNA and amplified for 18 cycles with the same kit as the R100 sample following the manufacturer’s protocol. The amplified cDNAs were sheared to ~380–base pair (bp) fragments with a Covaris M220 ultrasonicator (Covaris, Woburn, MA, USA), and Illumina libraries were prepared using the KAPA HyperPrep Kit (Kapa Biosystems, Wilmington, MA, USA). End repair and A-tailing reactions were carried out with 10.0 and 44.5 ng of input DNA, respectively. All steps to post-amplification cleanup were conducted according to the manufacturer’s instructions. Quantification of the libraries using a Bioanalyzer 2100 and KAPA library quantification kit revealed that more than 6.2 nM DNA was obtained from each library (table S1). The libraries were sequenced as 300-bp paired ends using the MiSeq v.3 600 cycles kit (Illumina Inc., San Diego, CA, USA) at Japan Agency for Marine-Earth Science and Technology, Yokosuka, Japan. The mRNA nucleotide sequences have been deposited in the National Center for Biotechnology Information (NCBI) Short Read Archive under accession numbers SRR17812219–SRR17812231 in biosample SAMN25377779 (*A. beccarii* T1/S4).

### De novo assembly and functional annotation

Sequence analysis was performed in CLC Genomics Workbench v8.6 (CLC bio, Cambridge, MA, USA) using default settings to remove adaptors and trim low-quality reads. The resulting high-quality reads were used for de novo assembly into scaffolds using default parameters and a minimum contig length of 300 bps. In total, ~48 million raw reads were generated from R100 and C1 libraries, and 38.6 million high-quality reads were used for de novo assembly into 54,914 scaffolds with an average length of 1039 bps and an N50 length of 1400 bps. The total length of the assembled scaffolds was ~57 Mbps, with an average GC content of 43.6%.

Scaffold sequences were applied to ORFs using the MetaGeneMark web server (http://exon.gatech.edu/meta_gmhmmp.cgi). The 48,542 ORFs exceeding 80 amino acids in length were functionally annotated using the Kyoto Encyclopedia of Genes and Genomes (KEGG) Automatic Annotation Server database with the single-directional best hit method set to a threshold assignment score of 37 (*61*), the Clusters of Orthologous Groups of proteins derived from prokaryotic and unicellular eukaryotic genome sequences (*62*–*64*), the Eukaryotic Orthologous Group derived from seven eukaryotic genomes (*64*), and the Pfam protein families database (*65*). Protein localization was predicted on the basis of transmembrane helices in the TMHMM server (version 2.0) (*66*). The taxonomic assignment of each ORF was analyzed in GhostKOALA (*67*).

### Mapping of raw reads to ORFs and differential gene expression

The reads of each RNA library were mapped against the 48,542 ORFs generated from R100 and C1 libraries using a length cutoff of 0.5 and a similarity fraction of 0.98 in the CLC Genomics Workbench v8.6 RNA-seq algorithm. RPKM mapped reads (*68*) were calculated separately for all single-cell RNA libraries and used to compare gene expression levels. Because the read numbers of the C1 library were extremely large (table S1), we randomly sampled 300 reads. A multidimensional scaling diagram was generated using the gene expression profiles of C1 to C9 and N1 to N3 libraries to visualize the relationship among them.

To visualize differences in expression across experimental conditions, we conducted a significance test for the read counts of each calcification condition (C1 to C9) against the average of control conditions (N1 to N3) using DESeq2 (*69*). Differential gene expression analysis yielded 7390 CDSs, significantly expressed (*P* < 0.05) during the calcification stage. In the absence of valid genomic information or transcriptomic data for Foraminifera, we relied on the KEGG pathways (*70*) and cell cycle observations in Rotaliida (*7*, *8*, *21*, *24*). According to the processes predicted during foraminiferal calcification (reticulopodia assembly, Ca^2+^ transport, and CaCO_3_ precipitation), we detected 27 genes, which showed significantly high expression in samples C1 to C9 (*P* < 0.05 in DESeq2), and an additional 29 genes associated with them (*P* > 0.05).

### Comparison of calcium-related genes among Rhizaria

We collected 39,963 protein-coding genes from published genome data of the naked basal foraminifer *R. filosa* (PRJNA29155). In addition, genome data of the endomyxans *Spongospora subterranea* (GCA_900404475) and *Plasmodiophora brassicae* (GCA_003833335) as well as that of the cercozoan *Bigelowiella natans* (GCA_000320545), which are members of Rhizaria, were collected from the NCBI database and used for ORF calling with TransDecoder v.5.5.0 (*71*). The protein family and domain of each ORF of *A. beccarii* and the other four reference species were assigned by HMMER v.3.3 search (*72*) based on the Pfam-A database (*47*). The Pfam database contained 316 families and domains categorized as calcium-related genes. All annotated calcium-related genes in each genome and transcripts of *A. beccarii* were summarized in table S3. The number of calcium-related genes was 4639 (*A. beccarii*), 3527 (*R. filosa*), 537 (*S. subterranea*), 782 (*P. brassicae*), and 1733 (*B. natans*). For a better understanding of foraminiferal evolution, Venn diagrams of calcium-related genes in *A. beccarii*, *R. filosa*, and averaged endomyxans and cercozoans were drawn with venneuler v.1.1.0 (*73*) in R. Each protein family or domain highly expressed during calcification was classified as “Cf-high,” and the relative abundance of Cf-high genes is reported in Fig. 6.

### Phylogenetic analysis of CA genes

We collected the binned genome data of the calcite test-forming foraminifer *Globobulimina* sp. (PIVH01) from the NCBI database and used it for ORF calling in the same manner as other endomyxan and cercozoan genomes. CA genes were classified into five families: α-CA, β-CA, γ-CA, δ-CA, and ζ-CA (*48*–*50*, *53*). CA genes in *A. beccarii*, *Globobulimina* sp., *R. filosa*, *S. subterranea*, *P. brassicae*, and *B. natans* were identified by BlastP using α-CA of *Klebsiella pneumoniae* (AAC77887.1), β-CA of *Escherichia coli* (P61517.1), γ-CA of *Pyrococcus horikoshii* (BAA30703.1), δ-CA of *Ostreococcus lucimarinus* (XP_001419453), and ζ-CA of *Prochlorococcus marinus* (WP_011132187.1) as queries at an *e* value of 1 × 10^−10^. Nucleotide sequences of the identified genes were translated into peptides using a universal genetic code and added to known alignments (*53*), including all five families of CA genes (all-CA dataset) (table S4). The all-CA dataset was aligned using MAFFT 7.427 (*74*), and the resulting alignments were curated using trimAl 1.2 (*75*) with the gappyout option. Maximum likelihood phylogenetic analysis in IQ-TREE 1.6.7 (*76*) was performed using WAG + R5 (*77*) (*78*) as the best fitting model chosen by ModelFinder (*79*). Node supports were obtained from ultrafast bootstrap approximation (*80*) with 1000 replicates and SH-aLRT (*81*) with 1000 bootstrap replicates. Additional maximum likelihood analysis was performed with RAxML-NG v.1.0.2 (*82*) under the best fitting model WAG + G4 (*83*) based on empirical codon frequencies chosen by ModelTest-NG (*84*), with 10 randomized parsimony start trees and node support obtained from 200 bootstraps. Bayesian analyses were run in PhyloBayes-MPI 1.7 (*85*) with the WAG + R model. Markov chain Monte Carlo (MCMC) runs, each with one “cold” and three “hot” chains, were performed in parallel, and the MaxDiff between both runs reached 0.3 after 1.8 × 10^9^ generations. These 1.8 × 10^8^ generations were discarded as burn-in, and the remaining trees were summarized to obtain Bayesian posterior probabilities (fig. S3).

For the α-CA and β-CA datasets, we chose sequences from all families of the all-CA dataset and collected CA genes from the KEGG GENOME database (*86*) to cover representative lineages of Archaea, Bacteria, and Eukaryota (table S4). We also searched the closest relative protein sequences from the nr database against CA genes of *A. beccarii* using BlastP and added them to the datasets. For the α-CA dataset, we added representative α-CA sequences categorized as membrane-associated/secreted, cytosolic/mitochondrial, and CA-related proteins (table S4) (*52*). Phylogenetic analyses of the α-CA and β-CA datasets were conducted in the same manner as those of the all-CA dataset. For the α-CA dataset, the best fitting models for IQ-TREE 1.6.7, RAxML-NG v.1.0.2, and PhyloBayes-MPI were LG + R6 (*87*), LG + G4, and LG + R, respectively. The MaxDiff of MCMC runs reached 0.3 after 8 × 10^8^ generations. Next, 8 × 10^7^ generations were discarded as burn-in, and the remaining trees were summarized to obtain Bayesian posterior probabilities. For the β-CA dataset, the best fitting models for IQ-TREE 1.6.7, RAxML-NG v.1.0.2, and PhyloBayes-MPI were LG + R6, LG + I + G4 (*88*), and LG + R, respectively. The MaxDiff of MCMC runs reached 0.3 after 5 × 10^8^ generations. Next, 5 × 10^7^ generations were discarded as burn-in, and the remaining trees were summarized to obtain Bayesian posterior probabilities (Fig. 8). For the α-CA and β-CA datasets, we predicted signal peptides using SignalP v.5.0b (*89*) and TargetP v.2.0 (*90*), mitochondria-targeting signals using MitoFates (*91*), and glycosylphosphatidylinositol anchors using GPI-SOM (*92*). Transmembrane sites were predicted using TMHMM v. 2.0 (*66*). LCRs of all sequences were predicted using SMART (*93*), and the theoretical pH of each LCR was calculated using ProtParam (*94*). All predictions are summarized in tables S5 to S9.

### Cytosolic calcium and pH imaging

The dynamics of intracellular calcium ions and pH were documented using the calcium indicator Rhod-3 AM (8 μM; Thermo Fisher Scientific) and pyranine (40 μM; 8-hydroxypyrene-1,3,6-trisulfonic acid) (*7*, *23*). *A. beccarii* specimens were incubated at ~23°C overnight in seawater medium containing both Rhod-3 AM (excitation: 550 nm and emission: 600 nm) and pyranine (excitation: 410/470 nm and emission: 535 nm). Time-lapse fluorescence images were obtained using a Zeiss Axio observer Z1 inverted microscope (Zeiss, Oberkochen, Germany) with differential interference contrast images. Time-lapse images (Fig. 3) were recorded at 2-min intervals. Time-lapse images (movie S1) were recorded at 3-min intervals for the first 36 min and then every 10 min. Time-lapse images were captured by a digital camera attached to the microscope using Zeiss AxioVision software v. 4.6. Images of Rhod-3 AM were acquired using an FS43HE filter set (excitation: 550/25 nm, Fourier transform (FT): 570, and emission: 605/70 nm) with fluorescence intensity in grayscale. The fluorescence images at two excitation wavelengths (λ410 and λ470 nm) were taken for pH ratiometric imaging. Filter factors were excitation: 410/30 nm, FT: 500, and emission: 535/50 nm for λ410 nm and excitation: 470/20 nm, FT: 500, and emission: 535/50 nm for λ470 nm. Images were documented in grayscale. The pH images were then calculated by dividing λ470exc by λ410exc for each image (*7*, *21*, *23*). Images were processed with Zeiss Zen 2.6 Pro software and displayed in pseudocolor.

## Notes

View/request a protocol for this paper from *Bio-protocol*.
