# Supplementary material for: Unique evolution of foraminiferal calcification to survive global changes
Source: Sci Adv. 2023 Jun 21;9(25):eadd3584. doi: 10.1126/sciadv.add3584 (PMC10284544; doi:10.1126/sciadv.add3584)
Supplement: Supplementary file 1 — Figs. S1 to S5 Legends for tables S1 to S9 Legend for movie S1 [file sciadv.add3584_sm.pdf]

Science Advances
AAAS

Supplementary Materials for

Unique evolution of foraminiferal calcification to survive global changes

Yurika Ujiié *et al.*

Corresponding author: Yurika Ujiié, yujiie@kochi-u.ac.jp

Sci. Adv. **9**, eadd3584 (2023)
DOI: 10.1126/sciadv.add3584

The PDF file includes:

Figs. S1 to S5
Legends for tables S1 to S9
Legend for movie S1

Other Supplementary Material for this manuscript includes the following:

Tables S1 to S9
Movie S1

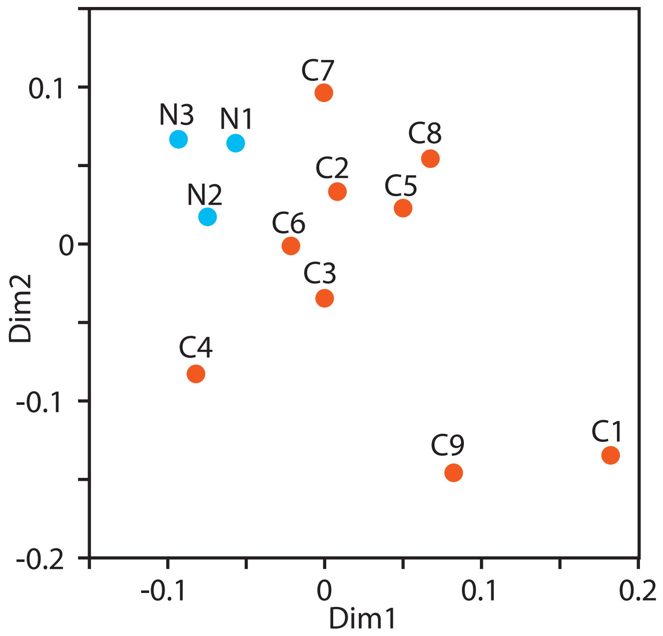

Fig. S1. Multidimensional scaling diagram comparing RNA-seq libraries obtained during control (N1–N3) and calcification (C1–C9) conditions.

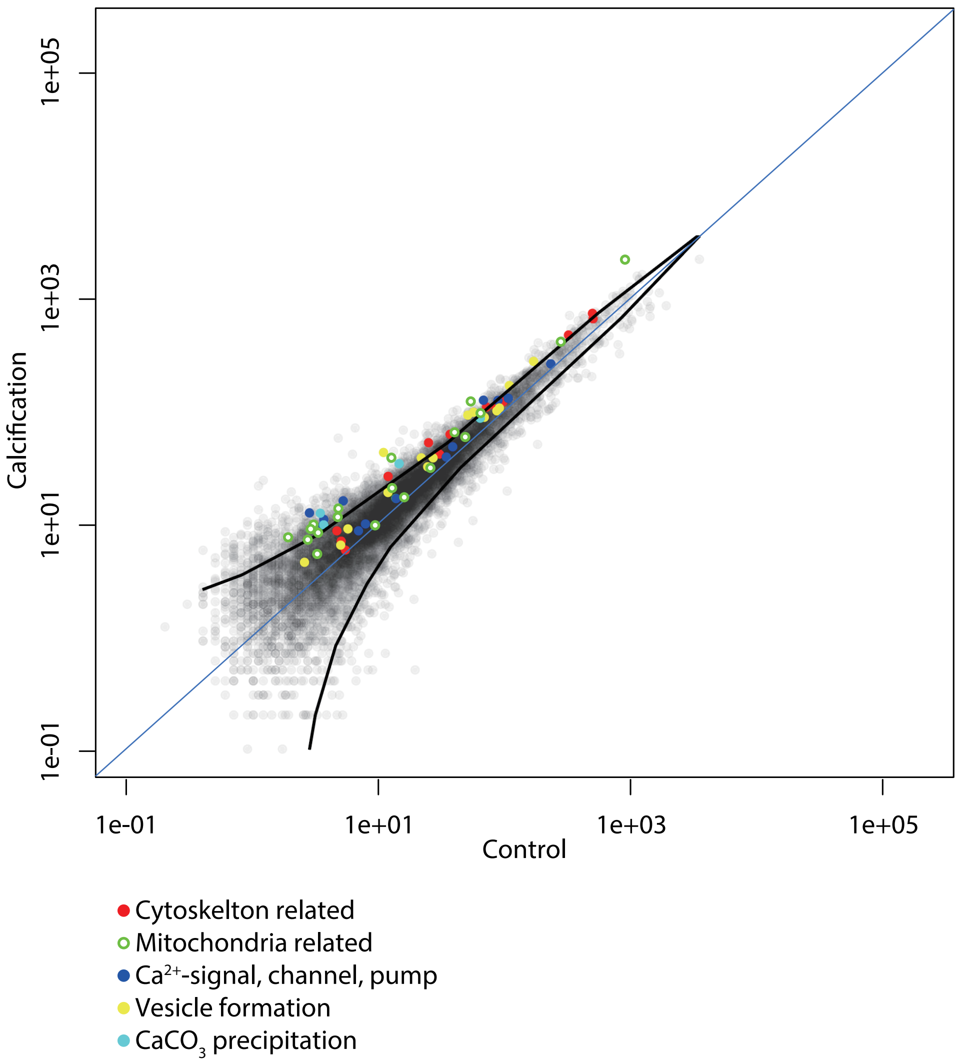

Fig. S2. Scatter plots of transcriptomes from calcification samples (C1–C9) and control samples (N1–N3). The black line indicates the $P = 0.05$ value in DESeq2. Unique colored points indicate genes associated with different cellular components or events during the calcification process: reticulopodia assembly (cytoskeleton- and mitochondria-related), Ca^{2+} transport, vesicle formation, and $CaCO_3$ precipitation, as detailed in Figs. 4 and 5.

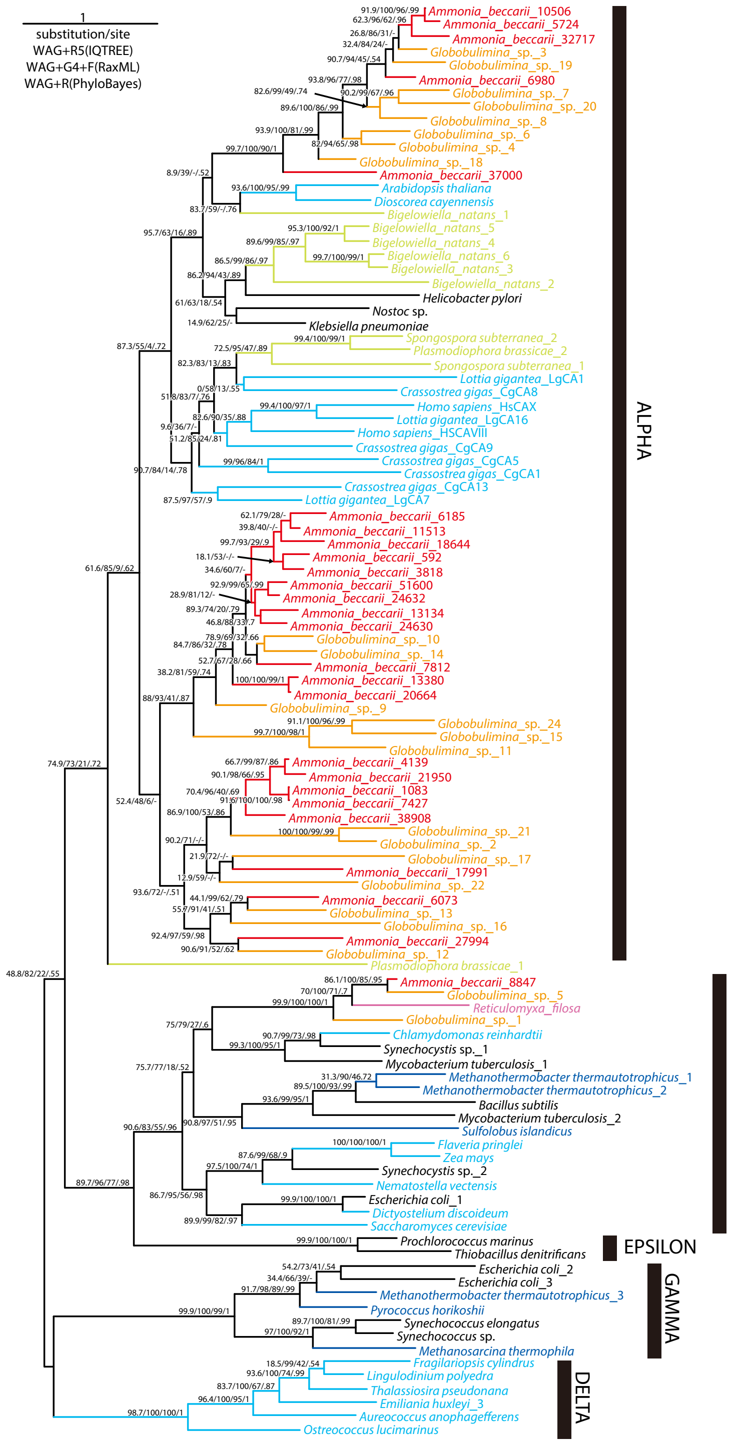

Fig. S3. Phylogeny of CA protein sequences. Phylogeny of representative sequences, including all CA families, showing the five clades corresponding to α-, β-, γ-, δ-, and ζ-CA families. Line colors indicate taxa: *A. beccarii* (red), *Globobulimina* sp. (orange), *R. filosa* (pink), cercozoan and endomyxans (light green), other eukaryotes (light blue), Archaea (blue), and Bacteria (black).

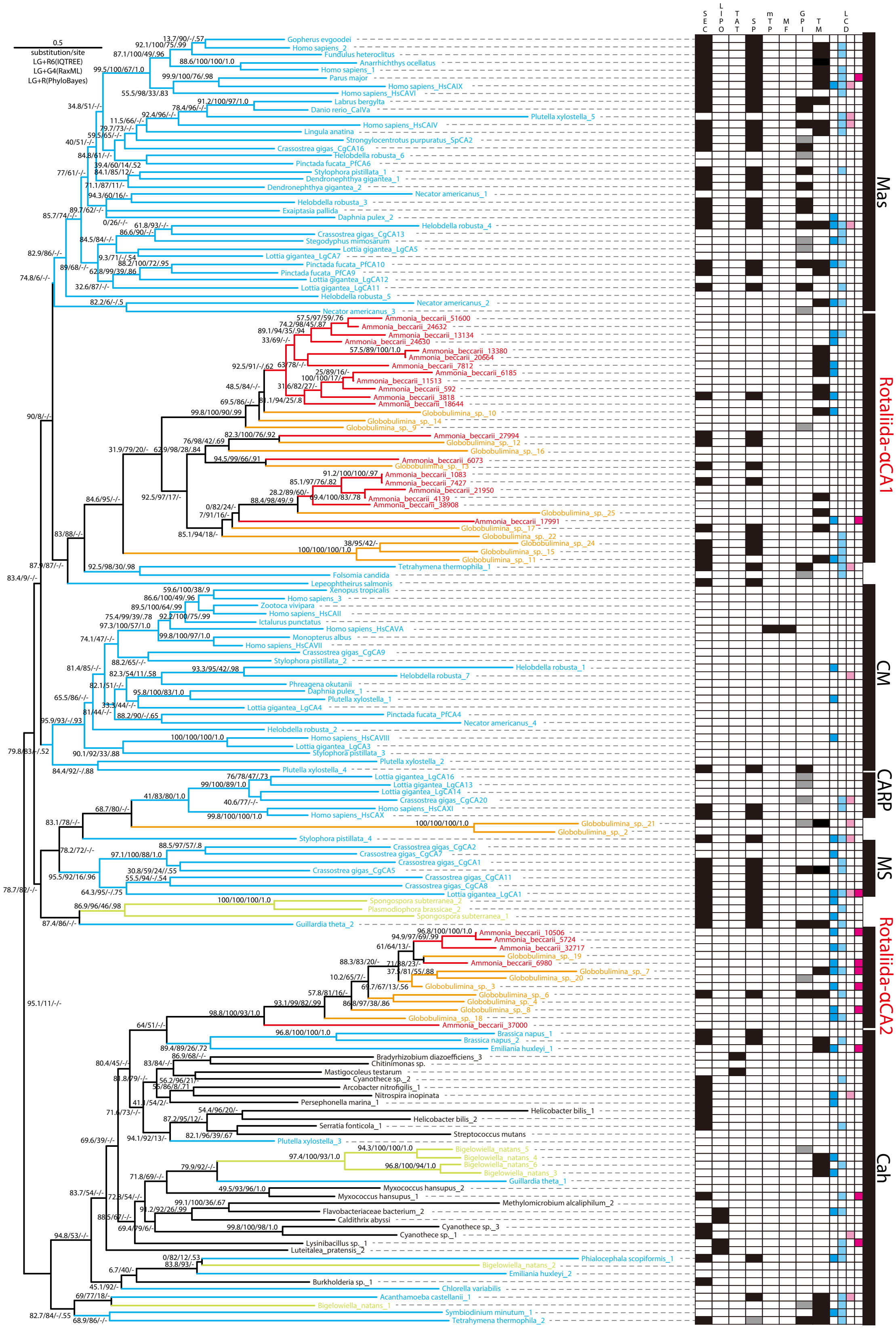

Fig. S4. **Phylogeny of α-CA protein sequences.** Line colors are same as Fig. S3.

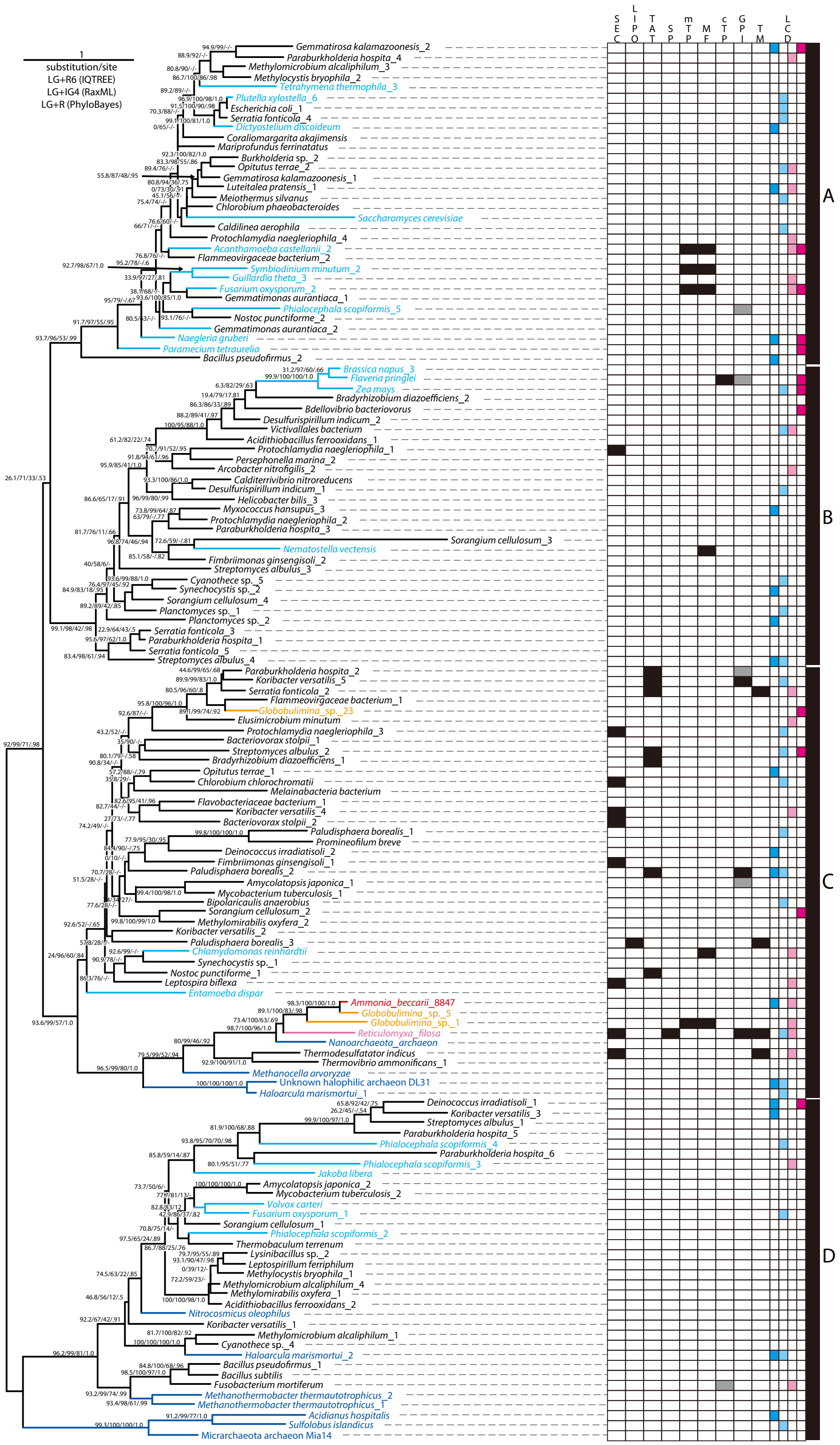

Fig. S5. Phylogeny of β-CA protein sequences. Line colors are same as Fig. S3.

Table S1 Sequence information regarding libraries obtained in this study.
Table S2 List of 56 genes and their RPKM value at each calcification condition (C1-C9) and control condition (N1-N3).
Table S3 Calcium-related proteins of foraminifers and cercozoans listed by domain-search of the Pfam database.
Table S4 List of OTU annotated as carbonic anhydrase.
Table S5 List of OTU and signal peptide predicted by SignalP_TargetP.
Table S6 List of OTU and mitochondria targeting predicted by MitoFates.
Table S7 List of OTU and glycosylphosphatidylinositol anchor signal predicted by GPISOM.
Table S8 List of OTU and transmembrane site predicted by TMHMM.
Table S9 List of OTU, low complexity region, and theoretical isoelectric point predicted by SMART_ProtParam.

Movie S1 Sequential time-lapse images of intracellular Ca^{2+} with the calcium indicator Rhod-3 AM.
